# A 2D qualitative movement assessment of a deceleration task detects football players with high knee joint loading

**DOI:** 10.1007/s00167-021-06709-2

**Published:** 2021-09-04

**Authors:** Stefano Di Paolo, Stefano Zaffagnini, Filippo Tosarelli, Fabrizio Aggio, Laura Bragonzoni, Alberto Grassi, Francesco Della Villa

**Affiliations:** 1 Department for Life Quality Studies QUVI, University of Bologna, Via Giulio Cesare Pupilli, 1, 40136 Bologna, BO Italy; 2 2nd Orthopaedic and Traumatologic Clinic, IRCCS Istituto Ortopedico Rizzoli, Bologna, Italy; 3 Department of Biomedical and Neuromotor Sciences, University of Bologna, Bologna, Italy; 4 Education and Research Department, Isokinetic Medical Group, FIFA Medical Centre of Excellence, Bologna, Italy

**Keywords:** ACL, 2D video analysis, ACL injury prevention, Return to sport, Deceleration, Soccer

## Abstract

**Purpose:**

The deceleration (pressing) is a common situational pattern leading to anterior cruciate ligament (ACL) injury in football. Although mainly assessed for performance purposes, a stronger focus on movement quality might support the screening of at-risk athletes. The aim of the present study was to describe a 2D scoring system for the assessment of the deceleration task and to associate it with the knee joint loading (knee abduction moment) evaluated through the gold standard 3D motion capture. The hypothesis was that lower 2D scores would be associated with higher knee joint loading.

**Methods:**

Thirty-four competitive football (soccer) players (age 22.8 ± 4.1, 16 females) performed a series of deceleration tasks. 3D motion analysis was recorded using ten stereophotogrammetric cameras, a force platform, and three high-speed cameras. The 2D qualitative assessment was performed via a scoring system based on the video analysis of frontal and lateral planes joint kinematics for five scoring criteria. The intra- and inter-rater reliabilities were calculated for each 2D scoring criteria. The peak knee abduction moment was extracted and grouped according to the results of the 2D evaluation.

**Results:**

An ICC > 0.94 was found for all the 2D scoring criteria, both for intra-rater and inter-rater reliability. The players with low 2D frontal plane scores and low total scores (0–4) showed significantly higher peak knee abduction moment values (*p* < 0.001). A significant negative rank correlation was found between the total score and the peak knee abduction moment (*ρ* = − 0.25, *p* < 0.001).

**Conclusions:**

The qualitative 2D scoring system described successfully discerned between athletes with high and low knee joint loading during a deceleration task. The application of this qualitative movement assessment based on a detailed and accurate scoring system is suitable to identify players and patients with high knee joint loading (high knee abduction moments) and target additional training in the scenario of the primary and secondary ACL injury risk reduction.

**Level of evidence:**

Level IV.

## Introduction

The anterior cruciate ligament (ACL) injury risk reduction is a current open question for the sports medicine community. The investigation of situational patterns that lead to ACL injury has gained importance in the light of primary and secondary injury prevention [13, 18, 19, 29]. Recent ACL injury video analysis studies [6, 11, 22] identified pressing/tackling as the most common situational patterns for ACL injury in football. The pressing pattern has a common frontal sprint with sudden deceleration close to the opponent. Although such movement task can be easily resembled in laboratory contexts, it has been mainly used for sprint performance purposes.

Few studies conducted a biomechanical investigation of the deceleration task [15, 25]. A recent prospective study by Dix et al. [15] found significant differences in lower limb biomechanics between injured and uninjured female basketball players, advocating the deceleration task as a valuable screening task for ACL injury.

To facilitate the screening for ACL injury risk in a user-friendly fashion, 2D scoring systems are emerging as a valuable option for complex movement assessment during rehabilitation after ACL injury and reconstruction [5, 12, 16, 23, 26, 28]. These scoring systems have been mainly focused on jump landings [5, 23, 26] and change of direction [12, 16, 28] tasks. To the best of the authors’ knowledge, no 2D assessment method of the deceleration task evaluation has been proposed. Given the intrinsic speed difference reached in a single frontal sprint with deceleration compared to a multidirectional task, the investigation of such a discussed pattern might have a significant role in primary and secondary ACL injury risk patients’ stratification and in the development of personalized preventative strategies.

Therefore, the aim of the present study was to describe a 2D scoring system for the qualitative assessment of a deceleration task and to look for potential associations with the knee abduction moment (KAM) evaluated through the gold standard 3D motion capture. The hypothesis was that lower 2D scores would be associated with higher KAM. A qualitative scoring system that accurately detects high knee joint loadings may be clinically relevant and useful to target additional training in the ACL injury.

## Materials and methods

The research study was approved by the Institutional Review Board (IRB approval: 555/2018/Sper/IOR of 12/09/2018) of Area Vasta Emilia Romagna Centro (AVEC, Bologna, Italy) and registered on ClinicalTrials.gov (Identifier: NCT03840551). All the subjects signed informed consent before starting the acquisition protocol. The rights of the subjects were protected.

The analysis was conducted in the Education and Research Department of the Isokinetic Medical Group in Bologna (Italy). Overall, 34 recreational and elite footballers were recruited for the study (Table 1). Inclusion criteria were age between 18 and 50 years old and Tegner activity level at least 7. Exclusion criteria were: (1) evidence of musculoskeletal disorders or functional impairment; (2) body mass index (BMI) > 35; (3) previous surgery to lower limbs; (4) cardiopulmonary or cardiovascular disorders; (5) inability to perform the required tasks.

**Table 1:** Demographics data

Number of subjects	34
Gender (M/F)	18/16
Dominant limb (R/L)	30/4
Age (year)	22.8 ± 4.1 (18–31)
Height (cm)	174.8 ± 10.2 (157–191)
Weight (Kg)	68.6 ± 12.7 (51–94)
BMI	22.6 ± 2.6 (18–27)
Tegner	8.6 ± 1.0 (7–9)

Data are expressed as mean ± standard deviation (range). Dominant limb is meant as the preferred one used to kick a ball

### Deceleration acquisition protocol

As part of a multi-movement assessment, each footballer performed a deceleration task. The athletes were asked to perform a 10 m frontal sprint at maximum speed possible followed by deceleration and a single support strike on a force platform, and a last backward sprint. Full foot contact on the force platform was required to consider a trial valid. Before the test, the athletes performed a 10 min dynamic warm-up and few repetitions of the movement to get confident with the environment and the motor task. A sport and exercise medicine physician specialized in sports biomechanics (F.D.V.) instructed each subject on the movements to perform and verified the validity of each trial. All athletes performed six valid repetitions (three right and three left strikes).

3D motion analysis was recorded in a specialized laboratory equipped with artificial turf [12, 14]. A set of ten stereophotogrammetric cameras, a force platform embedded in the floor (AMTI 400 × 600, Watertown, MA USA), and three high-speed cameras placed frontally and bilaterally towards movement direction (VICON Nexus, Vicon Motion Systems Ltd, Oxford, UK) were used. The sampling frequency of the cameras and the force platform was 120 Hz, while the sampling frequency of the high-speed cameras was 100 Hz. The systems were synchronized for direct data comparison.

The system calibration was performed at the beginning of the acquisition and repeated periodically during the session. A total of 42 retroreflective markers were placed on each subject according to the full-body Plug-in Gait protocol. The same expert user conducted the entire marker positioning process. After marker positioning, subjects’ model calibration was performed before each acquisition.

### Data processing—3D analysis

Joint kinematics, kinetics, and GRF were quantified through VICON Nexus software. Marker trajectories were collected through the stereophotogrammetric cameras and interpolated through a spline-based algorithm; GRFs were collected through the force platform. The KAM was quantified using the standard “bottom-up” inverse dynamics approach of the Plug-in Gait protocol. The entire phase of foot contact on the force platform was considered in the analysis. The peak KAM value was extracted for each trial and normalized by the subject’s body weight (BW).

### Data processing—2D analysis

A 2D scoring system was adopted based on the qualitative movement evaluation of frontal and sagittal plane joint kinematics. Such a scoring system is included in a clinical multiple movements evaluation for RTS decision-making after ACL reconstruction [7, 8, 12]. The test is aimed to identify biomechanical and neuromuscular control deficits providing an intuitive and quick response to the patient. The evaluation is performed in a specific VICON software environment through the recordings of the three high-speed cameras and the resultant GRF vector of the force platform. Joint kinematics are evaluated at the frame of maximal knee flexion angle after the foot contact with the force platform.

Each deceleration trial was evaluated through five scoring criteria [12], and a score from 0 to 2 was attributed to the movements for each scoring criteria.

### Statistical analysis

The intraclass correlation coefficient (ICC) was used to calculate intra-rater and inter-rater reliability for each criterion and the total score. The reliability ICC values lower than 0.50, between 0.50 and 0.75, between 0.75 and 0.90, and greater than 0.90 were considered poor, moderate, good, and excellent, respectively [21].

The peak KAM extracted from each trial was grouped according to the results of the 2D evaluation. The normal distribution of the data was verified through the Shapiro–Wilk test. The categorical variables were presented as a percentage over the total, while the continuous variables were presented as mean ± standard deviation (95% confidence interval–CI).

The association between the peak KAM and the two components of the limb stability (LS) criteria (i.e., the frontal plane knee projection angle–FPKPA and the GRF vector score [12]) was assessed. The peak KAM was decomposed in quartiles: the players with peak KAM higher than the third quartile were assigned to “high KAM” and the players with peak KAM lower than the first quartile were assigned to “low KAM”. The presence of outliers in the data was checked. Three groups were generated for the 2D parameters: LS 0, 1, 2; GRF vector score 0, 1, 2. The FPKPA was also divided into three groups based on the terciles: lower than the first tercile, between the first and the third tercile, higher than the third tercile. The rate of 0/2, 1/2, and 2/2 scores obtained by the players was computed according to the KAM quartiles. The Cochran’s *Q* test was used to assess the rate differences.

Furthermore, the peak KAM was grouped according to the LS, the FPKPA, the GRF vector score, and the Total score. The one-way ANOVA was used to investigate the statistical differences among the three groups, and the Student’s *t* test with the Bonferroni correction for multiple comparisons was used to investigate the differences between the single groups.

Spearman’s coefficient *ρ* was used to investigate the rank correlation between the 2D Total Score and the peak KAM.

Differences were considered statistically significant for *p* < 0.05. All the statistical analyses were performed in SPSS (v26, IBM, Armonk, NY, US).

An a-priori power-analysis was performed in G*Power (v3.1, Brunsbüttel, Germany) based on the results of a previous similar study analyzing the deceleration task [25]. Considering a standard deviation of 0.7 N/BW (Newton/Body Weight) and a mean group difference of 1 N/BW (Effect size 1.43), at least 20 subjects were required to have a power of 0.9 and a type I error of 0.05.

## Results

Overall, 181 valid trials were included in the analysis. The peak KAM was 1.4 ± 1.0 N/BW and 1.6 ± 0.8 N/BW for male and female athletes, respectively (n.s.). Both the intra-rater and inter-rater reliability showed an ICC between 0.94 and 1.00 for all the 2D criteria and total score. The first and the third terciles of the FPKPA were 21° and 33°, respectively. No outliers were present in the peak KAM data distribution.

### Peak KAM according to the 2D scores

Peak KAM significantly differed among the three groups based on the LS, the FPKPA, the GRF vector, and the Total score (*p* < 0.001) (Table 2, Appendix Fig. 3). The players with LS 0/2, FPKPA > 33°, GRF vector score 0/2, and Total score 0–4 showed significantly higher peak KAM values (Table 3). A significant negative rank correlation was found between the total score and the peak KAM (*ρ* = − 0.25, *p* < 0.001).

**Table 2:** Peak knee abduction moment based on the 2D scoring system

2D score	Groups			*p* value
LS	0	1	2	< 0.001*
	1.7 ± 1.0	1.2 ± 0.7	1.1 ± 0.8	
FPKPA	> 33°	21–33°	< 21°	< 0.001*
	1.9 ± 1.0	1.3 ± 0.8	1.2 ± 0.8	
GRF vector to knee	0	1	2	< 0.001*
	2.0 ± 0.9	1.4 ± 0.7	1.2 ± 0.8	
Total score	0–4	5–7	8–10	< 0.001*
	1.8 ± 1.0	1.4 ± 0.1	1.1 ± 0.9	

Data are expressed in Newton*m/Bodyweight as mean ± standard deviation

^*^Statistically significant differences between the three groups evaluated through the ANOVA (*p* < 0.05). “GRF vector to knee” means the knee-GRF vector overlay on the frontal plane [12]

**Table 3:** Multiple comparisons of the knee abduction moment based on the 2D evaluations

2D score	Diff (95% CI)	*p* value
LS		
0 vs 1	0.5 (0.1–0.8)	< 0.001*
0 vs 2	0.6 (0.1–1.1)	0.030*
1 vs 2	0.1 (− 0.5–0.6)	n.s
FPKPA		
> 33° vs 21–33°	0.7 (0.3–1.0)	< 0.001*
> 33° vs < 21°	0.8 (0.4–1.1)	< 0.001*
21–33° vs < 21°	0.1 (− 0.3–0.5)	n.s
GRF vector to knee		
0 vs 1	0.7 (0.2–1.1)	< 0.001*
0 vs 2	0.9 (0.5–1.2)	< 0.001*
1 vs 2	0.2 (− 0.2–0.6)	n.s
Total score		
0–4 vs 5–7	0.4 (0.0–0.8)	n.s
0–4 vs 8–10	0.8 (0.2–1.3)	< 0.001*
5–7 vs 8–10	0.4 (− 0.1–0.8)	n.s

Differences between the single groups are expressed in Newton*m/Bodyweight as mean (95% CI)

^*^Statistically significant differences between the single groups evaluated through the *t* test with Bonferroni correction (*p* < 0.05). “GRF vector to knee” means the knee-GRF vector overlay on the frontal plane [12]

### Rate of 2D score assigned to high- and low-peak KAM

**Fig. 1:**
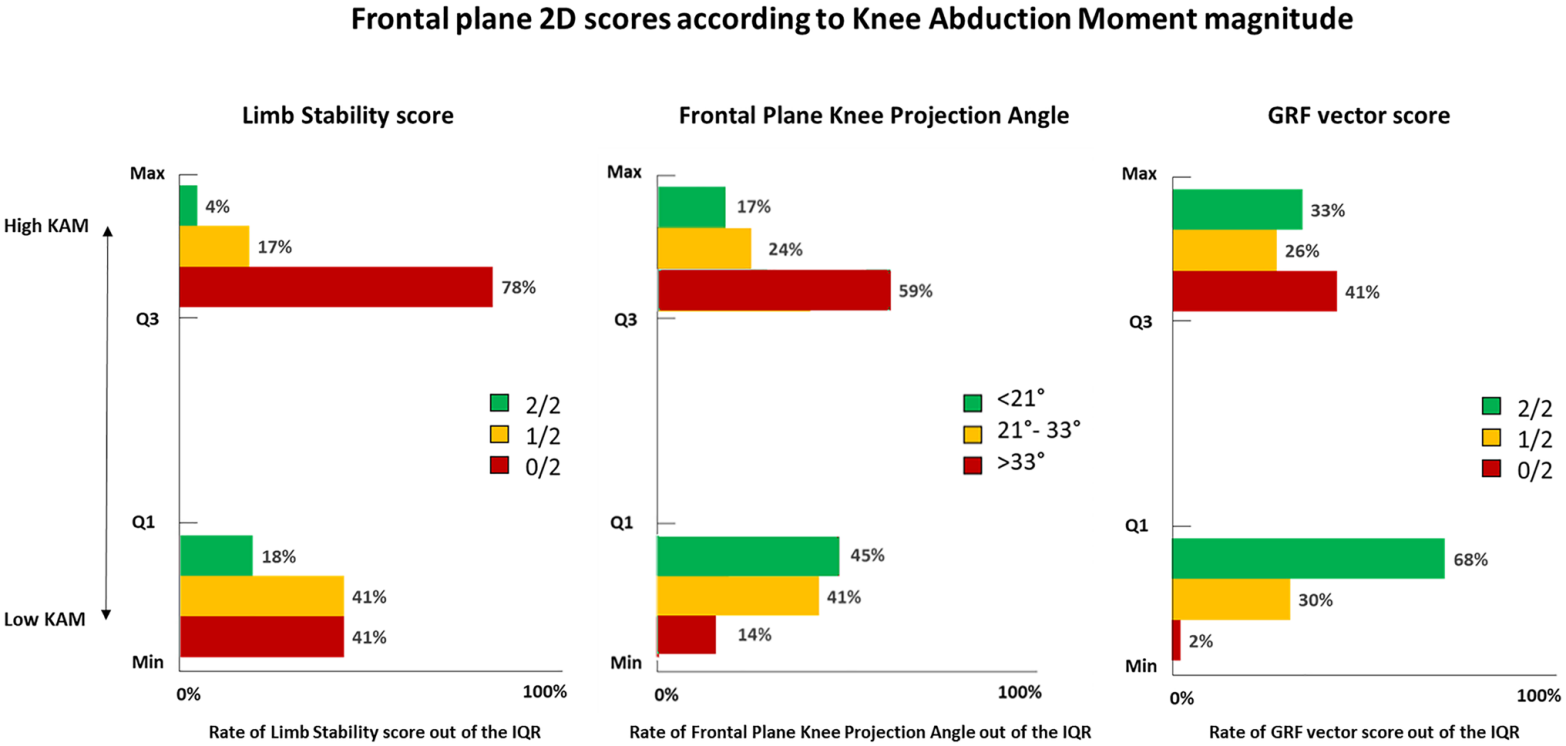
Rate of frontal plane 2D scores classified according to the peak knee abduction moment (KAM) values out of the interquartile range (IQR). High KAM and low KAM represent the values (N*m/BW) between third quartile and maximum and first quartile and minimum, respectively

The low KAM values ranged from − 0.4 N/BW to 0.8 N/BW (minimum-Q1) and the high KAM values ranged from 2.1 N/BW to 4.7 N/BW (Q3-maximum). The low KAM values corresponded to the better 2D scores, i.e., a higher percentage of FPKPA < 21° (45%, *p* < 0.001) and of GRF vector score 2/2 (68%, *p* < 0.001). The high KAM values corresponded to the worst 2D scores, i.e., a higher percentage of 0/2 score for the LS (78%, *p* < 0.001) and of FPKPA > 33° (59%, *p* < 0.001) (Fig. 1).

## Discussion

The main finding of the present study was the association between the 2D scoring system and the knee joint loading (KAM) in the biomechanical assessment of a deceleration task. Lower 2D scores were associated with higher KAM, thus confirming the study hypothesis. Both inter-and intra-rater reliability was excellent (ICC > 0.94) for the 2D criteria and total score. Such a qualitative 2D scoring system could, therefore, be a useful tool in ACL injury risk screening and prevention.

Despite the overwhelming evidence that pressing (therefore deceleration tasks) is a major situational pattern for ACL injury in football [6, 11, 18, 27], there is a lack of research on this kind of task. The research suggests that high-intensity engagements involving deceleration tasks are drawn into ACL injury causation in footballers. Nonetheless, a stronger focus should be paid to the quality of movement execution. The performance-injury conflict is largely debated in the sports medicine community. A recent investigation by Dos’Santos et al. highlighted that the most performant players adopt less safe biomechanical patterns in sport-specific movements [17]. The authors advocated the need for a broader consciousness on performance-injury conflict and for preventative screenings.

**Fig. 2:**
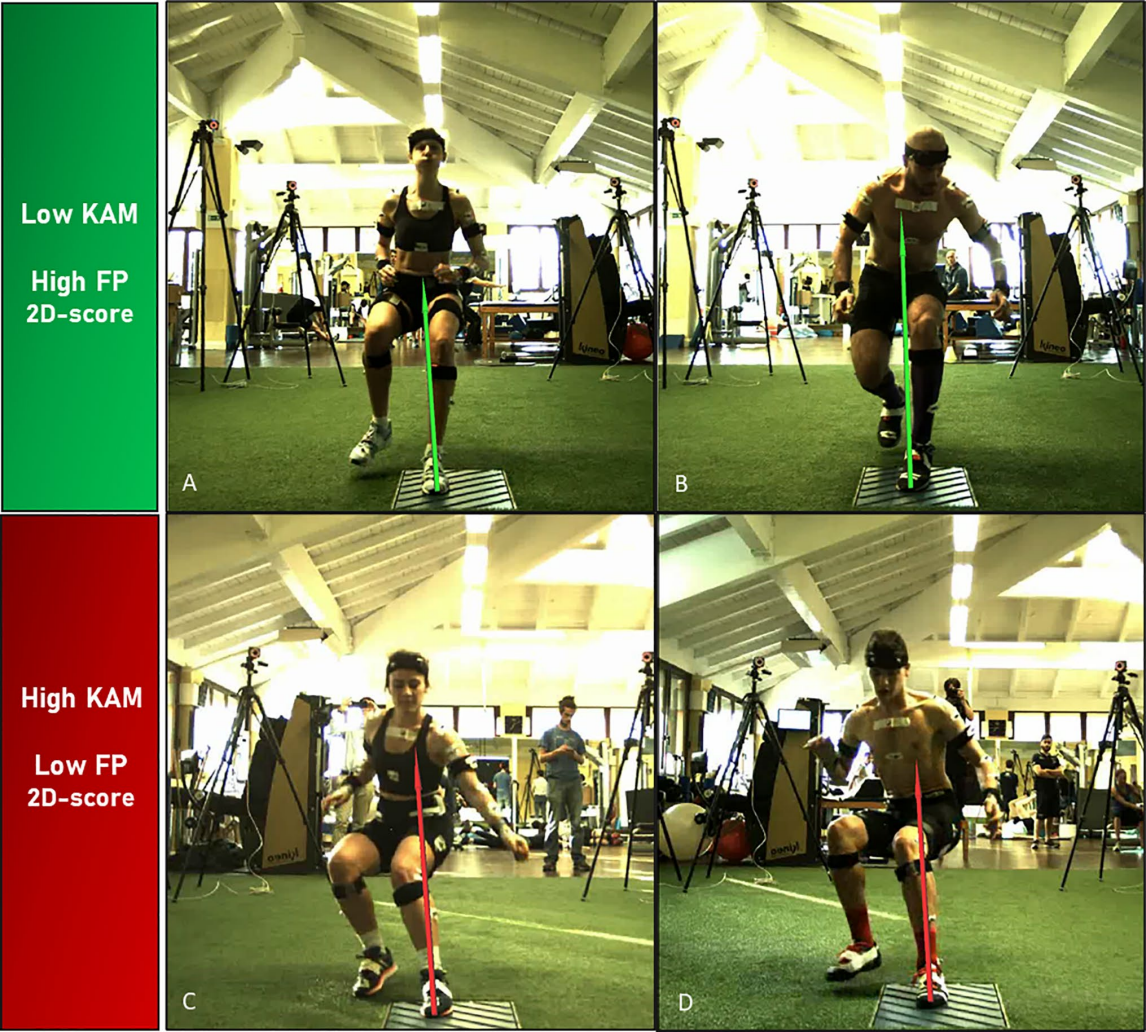
**A–B** Example of deceleration tasks performed with low knee abduction moment and corresponding high 2D score; **C–D** example of deceleration tasks performed with high knee abduction moment and corresponding low 2D score

As already demonstrated, KAM and medial knee motion during a deceleration task may predict an ACL injury [15]. In the present study, low 2D score both on frontal plane and considering the whole scoring system (Fig. 2) were associated with knee joint overloading (i.e., high KAM). The integrated frontal plane assessment (i.e., LS criteria, the FPKPA, and the GRF vector) demonstrated great sensibility and specificity for the KAM: only 4% of the athletes were misclassified in the high-KAM group and 2% in the low-KAM group (Fig. 1). Thus, a good level of accuracy has been reached despite the quick and cost-effective fashion. The total score was significantly correlated with the KAM. Furthermore, a total score between 0/10 and 4/10 was associated with an average 0.8 N*m/BW higher KAM compared to those obtaining at least 8/10. The latter finding supports the use of the 2D total score as a simple—but comprehensive—indication of an athlete’s movement quality in the deceleration task.

Such an evaluation may be applied to screen for additional preventative neuromuscular training, both in the primary and secondary prevention setting. Despite the open debate on the use of targeted neuromuscular training [4, 24], “at-risk” athletes were found to benefit more from additional customized programs focused on movement quality [20]. Given the growing incidence of ACL injuries in males and (recently) in females footballers [1, 3, 13], large-scale screenings and a broader awareness of the ACL injury risk are desirable.

Therefore, there are two main advantages of the qualitative movement assessment that we proposed. On one hand, it might provide detailed insights for ACL professionals on athlete’s biomechanics and potential ACL injury risk. On the other hand, it might provide quick feedback to the athlete regarding his/her movement quality and progress over the additional training or prolonged rehabilitation.

For the first time, a qualitative 2D scoring system has been described and validated for a deceleration task. Such an assessment might be part of a wider test battery, alongside jumping tasks (drop vertical jump, hops) and cutting maneuvers, to better discriminate safe and dangerous movement strategies across various injury risk situational patterns.

The present study has some limitations. First, the data were collected on healthy athletes during a single session. Therefore, it was not possible to provide the sensibility of the 2D scoring system in a test–retest condition after a preventative training program, nor in the discrimination between ACL-intact and ACL-reconstructed patients. Second, no muscle activation data were collected during the task execution. The integration of EMG-driven data might provide interesting insights into the interaction between athletes’ movement quality and muscular strength. Moreover, the investigation was conducted in a controlled laboratory environment. Therefore, despite the footballers were required to perform the tasks at their maximum speed, the external focus (e.g., ball, opponents) was limited. Future studies might consider inserting this evaluation in a real-world scenario. Indeed, recent studies on neuromuscular control in ACL-injured patients argued that neurocognitive alterations might happen during the situational patterns leading to an ACL injury [2, 9, 10]. Therefore, the introduction of additional focuses could help to discriminate between safe and unsafe return to the field. Last, only valid trials (i.e., entire foot strike on the force platform) were investigated. The analysis of the non-valid trials might be considered in future studies for a wider comprehension of players’ movement quality in real scenarios.

The proposed qualitative assessment of a deceleration task could be introduced in a test battery for the primary and secondary ACL injury risk stratification alongside with most common movements, e.g., single- and double-leg hops and cutting maneuvers. In the day-by-day clinical practice, professionals might benefit from an accurate and cost-effective tool to investigate athletes’ movement quality during a sport-specific situational pattern frequently found in ACL injury causation. Such screening could promote the indication for movement re-training and targeted neuromuscular training both in primary and secondary ACL injury prevention.

## Conclusion

The qualitative 2D scoring system here described successfully identified football players with high KAM during a deceleration task. The application of this qualitative movement assessment based on a detailed and accurate scoring system is suitable to identify players and patients with high KAM (high knee abduction moments) and target additional training in the scenario of the primary and secondary ACL injury risk reduction.

## Figures and Tables

**Fig. 3:**
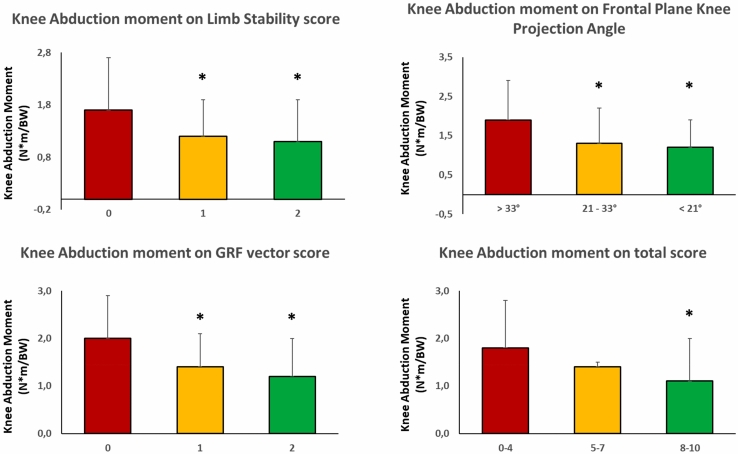
Knee abduction moment (KAM) based on the 2D scoring criteria: limb stability, frontal plane knee projection angle, ground reaction force (GRF) vector, total score. Bars with asterisk represent statistically significant differences (*p* < 0.05) from the lowest scored group (i.e., 0, > 33°, 0–4)

## Appendix

See Fig. 3

## Abbreviations

ACL: Anterior cruciate ligament
BMI: Body mass index
BW: Body weight
FPKPA: Frontal plane knee projection angle
GRF: Ground reaction force
ICC: Intraclass correlation coefficient
IQR: Interquartile range
KAM: Knee abduction moment
LS: Limb stability

## References

[1] Allen MM, Pareek A, Krych AJ, Hewett TE, Levy BA, Stuart MJ, Dahm DL (2016). Are female soccer players at an increased risk of second anterior cruciate ligament injury compared with their athletic peers?. Am J Sports Med.

[2] Anand M, Diekfuss JA, Slutsky-Ganesh AB, Grooms DR, Bonnette S, Barber Foss KD, DiCesare CA, Hunnicutt JL, Myer GD (2021). Integrated 3D motion analysis with functional magnetic resonance neuroimaging to identify neural correlates of lower extremity movement. J Neurosci Methods.

[3] Ardern CL, Ekås GR, Grindem H, Moksnes H, Anderson A, Chotel F, Cohen M, Forssblad M, Ganley TJ, Feller JA, Karlsson J, Kocher MS, LaPrade RF, McNamee M, Mandelbaum B, Micheli L, Mohtadi NGH, Reider B, Roe JP, Seil R, Siebold R, Silvers-Granelli HJ, Soligard T, Witvrouw E, Engebretsen L (2018). Prevention, diagnosis and management of paediatric ACL injuries. Br J Sports Med.

[4] Bahr R (2016). Why screening tests to predict injury do not work-and probably never will: a critical review. Br J Sports Med.

[5] Bates NA, Myer GD, Hale RF, Schilaty ND, Hewett TE (2020). Prospective frontal plane angles used to predict ACL strain and identify those at high risk for sports-related ACL injury. Orthop J Sports Med.

[6] Brophy RH, Stepan JG, Silvers HJ, Mandelbaum BR (2015). Defending puts the anterior cruciate ligament at risk during soccer: a gender-based analysis. Sports Health.

[7] Buckthorpe M (2019). Optimising the late-stage rehabilitation and return-to-sport training and testing process after ACL reconstruction. Sports Med.

[8] Buckthorpe M, Della Villa F, Della Villa S, Roi GS (2019). On-field rehabilitation part 1: 4 pillars of high-quality on-field rehabilitation are restoring movement quality, physical conditioning, restoring sport-specific skills, and progressively developing chronic training load. J Orthop Sports Phys Ther.

[9] Criss CR, Melton MS, Ulloa SA, Simon JE, Clark BC, France CR, Grooms DR (2021). Rupture, reconstruction, and rehabilitation: a multi-disciplinary review of mechanisms for central nervous system adaptations following anterior cruciate ligament injury. Knee.

[10] Criss CR, Onate JA, Grooms DR (2020). Neural activity for hip-knee control in those with anterior cruciate ligament reconstruction: a task-based functional connectivity analysis. Neurosci Lett.

[11] Della Villa F, Buckthorpe M, Grassi A, Nabiuzzi A, Tosarelli F, Zaffagnini S, Della Villa S (2020). Systematic video analysis of ACL injuries in professional male football (soccer): injury mechanisms, situational patterns and biomechanics study on 134 consecutive cases. Br J Sports Med.

[12] Della Villa F, Di Paolo S, Santagati D, Della Croce E, Lopomo NF, Grassi A, Zaffagnini S (2021). A 2D video-analysis scoring system of 90° change of direction technique identifies football players with high knee abduction moment. Knee Surg Sports Traumatol Arthrosc.

[13] Della Villa F, Hägglund M, Della Villa S, Ekstrand J, Waldén M (2021). High rate of second ACL injury following ACL reconstruction in male professional footballers: an updated longitudinal analysis from 118 players in the UEFA Elite Club Injury Study. Br J Sports Med.

[14] Di Paolo S, Lopomo NF, Della Villa F, Paolini G, Figari G, Bragonzoni L, Grassi A, Zaffagnini S (2021). Rehabilitation and return to sport assessment after anterior cruciate ligament injury: quantifying joint kinematics during complex high-speed tasks through wearable sensors. Sensors.

[15] Dix C, Arundale A, Silvers-Granelli H, Marmon A, Zarzycki R, Snyder-Mackler L (2020). biomechanical measures during two sport-specific tasks differentiate between soccer players who go on to anterior cruciate ligament injury and those who do not: a prospective cohort analysis. Int J Sports Phys Ther.

[16] Dos’Santos T, McBurnie A, Donelon T, Thomas C, Comfort P, Jones PA (2019). A qualitative screening tool to identify athletes with “high-risk” movement mechanics during cutting: the cutting movement assessment score (CMAS). Phys Ther Sport.

[17] Dos’Santos T, Thomas C, McBurnie A, Comfort P, Jones PA (2021). Biomechanical determinants of performance and injury risk during cutting: a performance-injury conflict?. Sports Med.

[18] Grassi A, Smiley SP, Roberti di Sarsina T, Signorelli C, Marcheggiani Muccioli GM, Bondi A, Romagnoli M, Agostini A, Zaffagnini S (2017). Mechanisms and situations of anterior cruciate ligament injuries in professional male soccer players: a YouTube-based video analysis. Eur J Orthop Surg Traumatol.

[19] Hewett TE, Bates NA (2017). Preventive biomechanics: a paradigm shift with a translational approach to injury prevention. Am J Sports Med.

[20] Hewett TE, Ford KR, Xu YY, Khoury J, Myer GD (2017). Effectiveness of neuromuscular training based on the neuromuscular risk profile. Am J Sports Med.

[21] Koo TK, Li MY (2016). A guideline of selecting and reporting intraclass correlation coefficients for reliability research. J Chiropr Med.

[22] Lucarno S, Zago M, Buckthorpe M, Grassi A, Tosarelli F, Smith R, Della Villa F (2021). Systematic video analysis of anterior cruciate ligament injuries in professional female soccer players. Am J Sports Med.

[23] Padua DA, DiStefano LJ, Beutler AI, de la Motte SJ, DiStefano MJ, Marshall SW (2015). The landing error scoring system as a screening tool for an anterior cruciate ligament injury-prevention program in elite-youth soccer athletes. J Athl Train.

[24] Paterno MV, Kiefer AW, Bonnette S, Riley MA, Schmitt LC, Ford KR, Myer GD, Shockley K, Hewett TE (2015). Prospectively identified deficits in sagittal plane hip-ankle coordination in female athletes who sustain a second anterior cruciate ligament injury after anterior cruciate ligament reconstruction and return to sport. Clin Biomech (Bristol, Avon).

[25] Peel SA, Schroeder LE, Sievert ZA, Weinhandl JT (2019). Comparing anterior cruciate ligament injury risk variables between unanticipated cutting and decelerating tasks. J Appl Biomech.

[26] Poston GR, Schmitt LC, Ithurburn MP, Hugentobler JA, Thomas S, Paterno MV (2021). Reduced 2-D frontal plane motion during single-limb landing is associated with risk of future anterior cruciate ligament graft rupture after anterior cruciate ligament reconstruction and return to sport: a pilot study. J Orthop Sports Phys Ther.

[27] Waldén M, Krosshaug T, Bjørneboe J, Andersen TE, Faul O, Hägglund M (2015). Three distinct mechanisms predominate in non-contact anterior cruciate ligament injuries in male professional football players: a systematic video analysis of 39 cases. Br J Sports Med.

[28] Weir G, Alderson J, Smailes N, Elliott B, Donnelly C (2019). A reliable video-based ACL injury screening tool for female team sport athletes. Int J Sports Med.

[29] Weitz FK, Sillanpää PJ, Mattila VM (2020). The incidence of paediatric ACL injury is increasing in Finland. Knee Surg Sports Traumatol Arthrosc.

